# Patient and Proxy Recall After Providing Written or Oral Informed Consent to Participate in an Interventional Trial

**DOI:** 10.1001/jamanetworkopen.2022.14052

**Published:** 2022-05-13

**Authors:** Angela Huttner, Elodie von Dach, Virginie Prendki, Stephan Harbarth, Laurent Kaiser

**Affiliations:** 1 Division of Infectious Diseases, Geneva University Hospitals and Faculty of Medicine, Geneva, Switzerland; 2 Center for Clinical Research, Geneva University Hospitals and Faculty of Medicine, Geneva, Switzerland; 3 Department of Rehabilitation and Geriatrics, Geneva University Hospitals and Faculty of Medicine, Geneva, Switzerland; 4 Infection Control Program, Geneva University Hospitals and Faculty of Medicine, Geneva, Switzerland

## Abstract

This cohort study assesses recall rates among patients and their proxies who consented to participate in a randomized clinical trial.

## Introduction

Patients’ understanding and recall after granting written consent for trial participation are known to be suboptimal.^1^ A 2006 study among 44 hospitalized patients providing written consent to an interventional trial found that only 68% remembered the purpose of the trial 10 days later; one-fifth had no recollection of having consented to any study.^2^ Recall by patients granting oral consent, and by the healthy proxies granting written consent for patients without capacity, is underreported. We compared recall rates after 30 days for participation in a randomized clinical trial (RCT) among patients with capacity who had given written or oral consent and for proxies of patients without capacity who had given written consent.

## Methods

The RCT and this nested cohort study were approved by the Geneva Cantonal Ethics Commission. All participants or their proxies provided informed consent. This study is reported following the Strengthening the Reporting of Observational Studies in Epidemiology (STROBE) reporting guideline.

This nested prospective cohort study included all patients with capacity and all proxies of patients without capacity participating at a single site (Geneva University Hospital, Switzerland) of a multicenter RCT by von Dach et al^3^ comparing antibiotic durations for gram-negative bacteremia in adults hospitalized between 2017 and 2019. In line with Swiss law, consent could be granted in writing by the patient, orally with a witness’s signature (for patients who were illiterate or physically unable to sign), or in writing by the proxy of a patient without capacity.

Our primary outcome was the 30-day recall rate for RCT participation among patients providing written or oral consent and proxies providing written consent. On day 30, patients and proxies were contacted for the RCT’s first clinical follow-up. They were asked whether they remembered (1) that they were (or their dependent was) participating in the RCT, (2) that they had granted consent, (3) the RCT’s purpose, and (4) potential risks.

Data were analyzed in Stata statistical software version 16.0 (StataCorp). Comparisons were performed with Fisher exact test; associations were 2-sided with *P* = .05 considered significant. Univariate logistic regression was used to assess factors potentially associated with recall. Data were analyzed from November 1 to 30, 2021.

## Results

The Geneva site enrolled 240 patients in the RCT. Consent was granted in writing by 167 patients (69%), orally by 16 patients (7%), and in writing by the proxies of 57 patients (24%) without capacity. At 30 days, data were available for 231 patients (96%): 4 patients (2%) had died, 3 patients (1%) had been otherwise lost to follow-up, and 2 patients (1%) were not asked the nested study’s questions. Median (IQR) patient age was 83 (74-89) years; 157 patients (65%) were women (Table 1). All proxies were next of kin. The time spent presenting the study and whether participants and proxies asked questions are detailed in Table 1.

**Table 1.** Baseline Demographic Characteristics and 30-Day Recall By Patients and Proxies After Granting Consent to Participate

Measure	No. (%)				*P* value^a^	*P* value^b^
	All (N = 240)	Written consent (n = 167)	Consent by proxy (n = 57)	Oral consent (n = 16)		
**Baseline demographics of included patients**						
Sex						
Men	83 (35)	57 (34)	21 (37)	5 (31)	.75	>.99
Women	157 (65)	110 (66)	36 (63)	11 (69)		
Age, median (IQR), y	83 (74-89)	82 (71-88)	86 (81-91)	84 (72-91)	.001	.28
Time spent, mean (SD), min	23.6 (12.5)	21.5 (11.2)	27.9 (13.9)	30.9 (13.6)	<.001	.002
**Recall at 30 d** ^c^						
Do you know that you are (or your loved one is) participating in this study?						
Yes	156 (68)	111 (69)	36 (64)	9 (64)	.51	.77
No	75 (32)	50 (31)	20 (36)	5 (36)		
Missing	9 (4)	6 (4)	1 (2)	2 (13)	NA	NA
Do you remember granting consent for participation in this study?						
Yes	145 (63)	101 (63)	35 (63)	9 (64)	>.99	>.99
No	86 (37)	60 (37)	21 (37)	5 (36)		
Missing	9 (4)	6 (4)	1 (2)	2 (13)	NA	NA
Do you remember the purpose of this study?						
Yes	65 (28)	40 (25)	20 (36)	5 (36)	.12	.36
No	166 (72)	121 (75)	36 (64)	9 (64)		
Missing	9 (4)	6 (4)	1 (2)	2 (13)	NA	NA
Do you remember the risks of the study?						
Yes	18 (8)	10 (6)	5 (9)	3 (21)	.54	.07
No	213 (92)	151 (94)	51 (91)	11 (79)		
Missing	9 (4)	6 (4)	1 (2)	2 (13)	NA	NA

Abbreviation: NA, not applicable.

^a^ Comparison between written consent by patient and written consent by proxy (Fisher exact).

^b^ Comparison between written consent and oral consent by patient (Fisher exact).

^c^ Questions are presented as they were asked of the participant or proxy on day 30.

A total of 111 of 161 patients providing written consent (69%), 9 of 14 patients providing oral consent (64%), and 36 of 56 proxies (64%) remembered that they or their loved ones were participating in a trial. Furthermore, 60 patients providing written consent (37%), 5 patients providing oral consent (36%), and 21 proxies (37%) recalled granting consent; 40 patients providing written consent (25%), 5 patients providing oral consent (36%), and 20 proxies (36%) remembered the purpose of the trial. Few remembered the trial’s potential risks (Table 1). In linear and univariate regression models, neither the time spent with patients or proxies, whether they had questions, nor consent modality was associated with improved recall (Table 2).

**Table 2.** Factors Assessed for Potential Association With Recall of Participation in the Trial, by Linear Regression and Univariate Logistic Regression

Factor	Odds ratio (95% CI)	*P* value
Time spent with patient or proxy, regression coefficient (95% CI)	−0.0033 (−0.0082 to 0.0017)	.20
Patient or proxy asked questions	1.17 (0.62 to 2.20)	.63
Written consent by proxy	0.90 (0.65 to 1.24)	.52
Oral consent by patient	0.81 (0.26 to 2.54)	.72

## Discussion

This cohort study among hospitalized patients, most of whom were elderly and all of whom had been acutely ill and hospitalized in the days prior, found that most patients had poor recall of their written consent to participate in an interventional trial (63%), a finding consistent with earlier studies.^1,2^ Yet recall after oral consent was no worse (64%), suggesting that the act of signing a document was not associated with improved retention or understanding. Perhaps most strikingly, recall by proxies, presumably healthy, providing written consent for their loved ones was as poor as that of patients who were seriously ill (63%). This study is limited by the impossibility of randomizing candidates or proxies to oral or written consent and by the small number of patients granting oral consent.

The ability of patients—deemed competent by their physicians—to grant truly informed consent has long been in question.^4,5^ The ability of their proxies, physically healthy but emotionally stressed, to do the same requires further exploration. While these results require confirmation in larger studies, the act of signing consent, as opposed to granting it orally, was not associated with later recall or understanding.
